# Supplementary material for: Revealing the hidden structure of disordered materials by parameterizing their local structural manifold
Source: Nat Commun. 2024 May 24;15:4424. doi: 10.1038/s41467-024-48449-0 (PMC11126625; doi:10.1038/s41467-024-48449-0)
Supplement: Supplementary file 1 — Supplementary Information [file 41467_2024_48449_MOESM1_ESM.pdf]

Supplementary Information for Revealing the Hidden Structure of Disordered Materials by Parameterizing their Local Structural Manifold

Thomas J. Hardin[1], Michael Chandross[1], Rahul Meena[2], Spencer Fajardo[3], Dimitris Giovanis[2,8], Ioannis Kevrekidis[4,5], Michael Falk[3,6,7,8], and Michael D. Shields[2,3,8]

[1] *Material, Physical, and Chemical Sciences Center, Sandia National Laboratories*
[2] *Department of Civil and Systems Engineering, Johns Hopkins University*
[3] *Department of Materials Science and Engineering, Johns Hopkins University*
[4] *Department of Applied Mathematics and Statistics, Johns Hopkins University*
[5] *Department of Chemical and Biomolecular Engineering, Johns Hopkins University*
[6] *Department of Mechanical Engineering, Johns Hopkins University*
[7] *Department of Physics and Astronomy, Johns Hopkins University*
[8] *Hopkins Extreme Materials Institute, Johns Hopkins University*

January 15, 2024

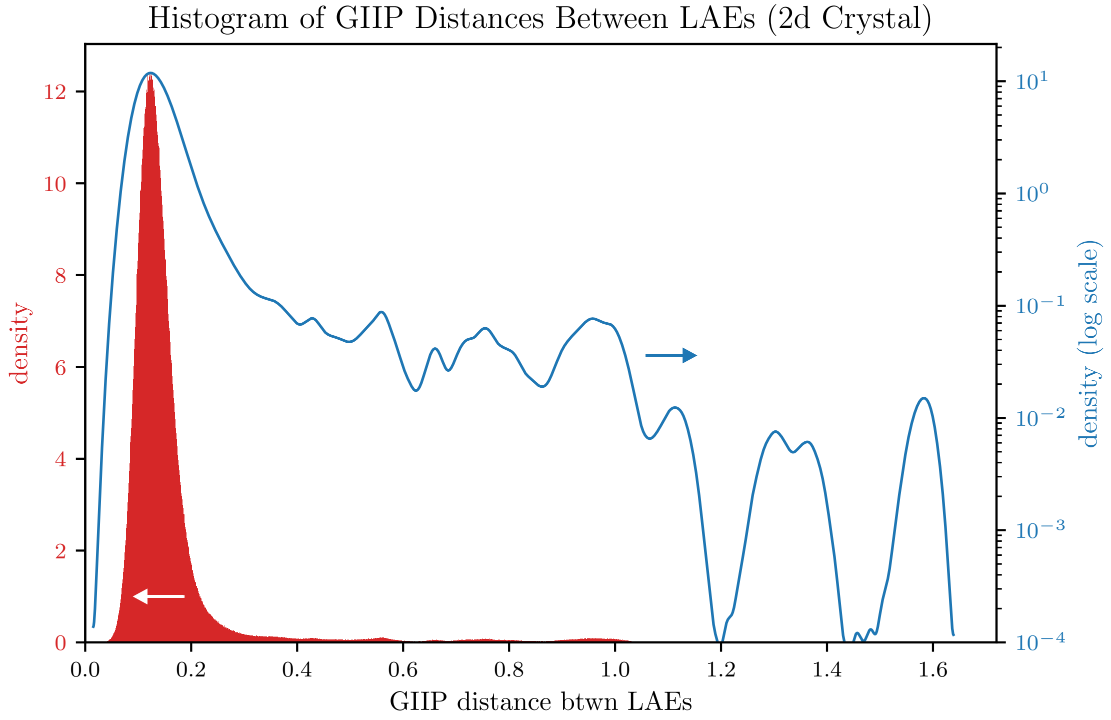

Supplementary Figure 1: Histogram of Gaussian Integral Inner Product (GIIP) distances between Local Atomic Environments (LAEs) in 2d crystal sample. The primary peak reflects distances between "perfect crystal" LAEs, while shorter, more distant peaks are indicative of distinct defect classes. Source data are provided as a Source Data file.

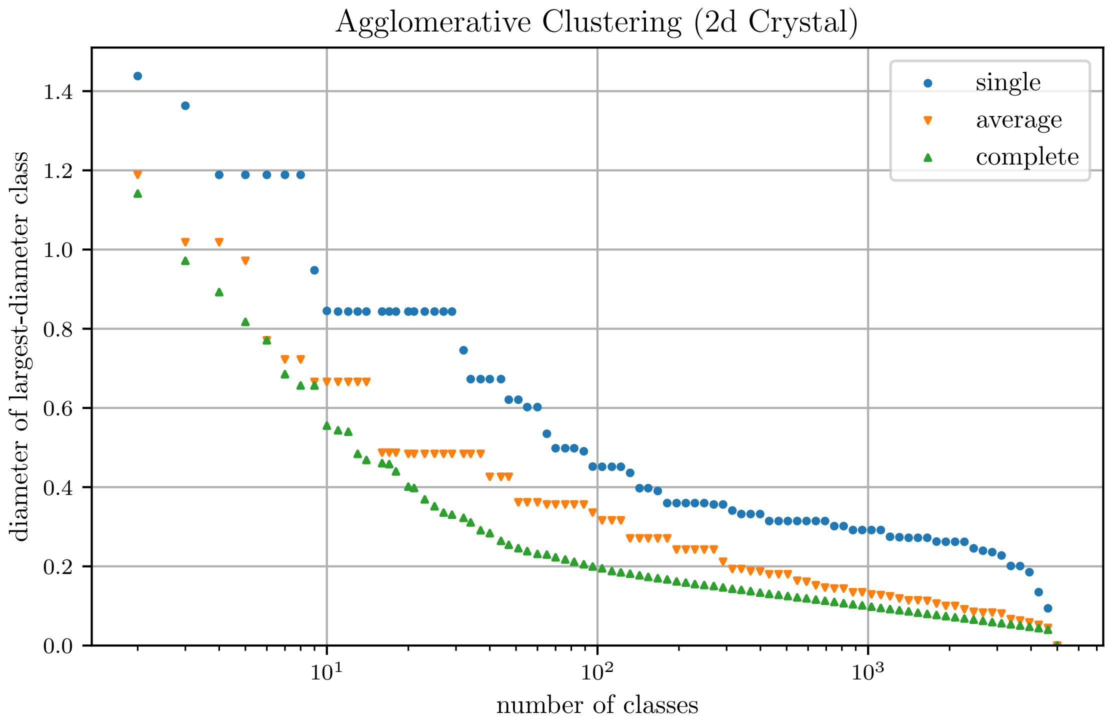

Supplementary Figure 2: Diameter of largest-diameter class as a function of number of classes for the 2d crystal sample. Diameter of a class is the largest GIIP distance between members of the class. Only a few classes are needed to achieve a small amount of within-class variation. Source data are provided as a Source Data file.

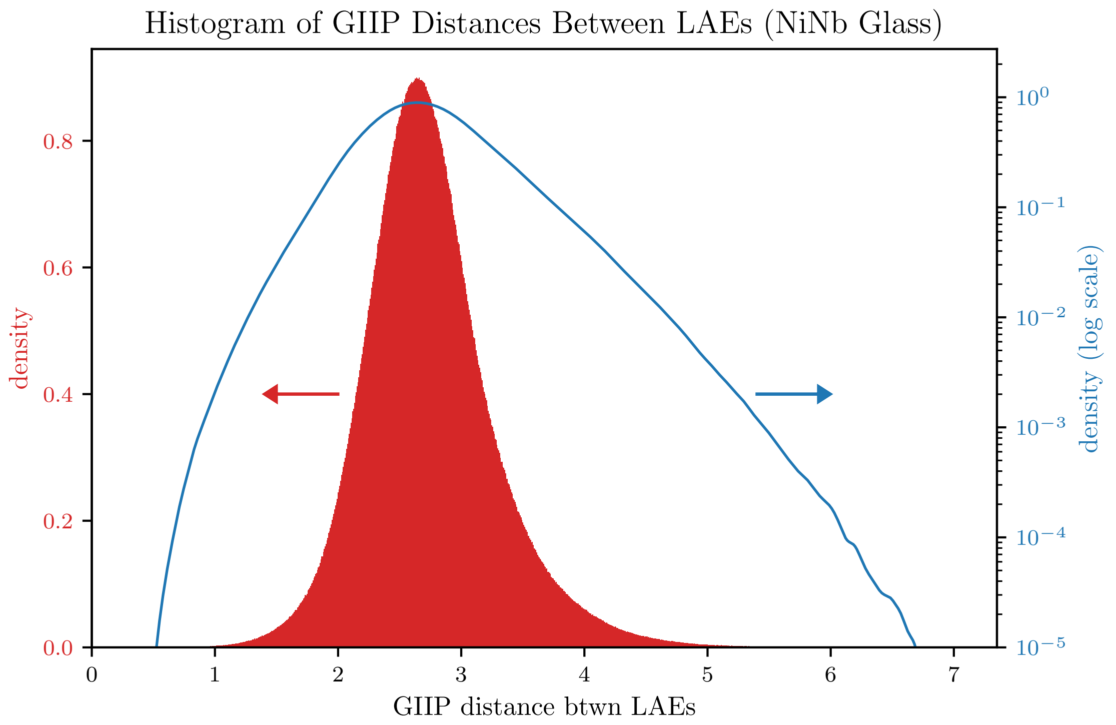

Supplementary Figure 3: Histogram of GIIP distances between LAEs in 3d NiNb glass sample. The smooth single-peaked distribution reflects the large number of distinct LAEs present in the glass. Source data are provided as a Source Data file.

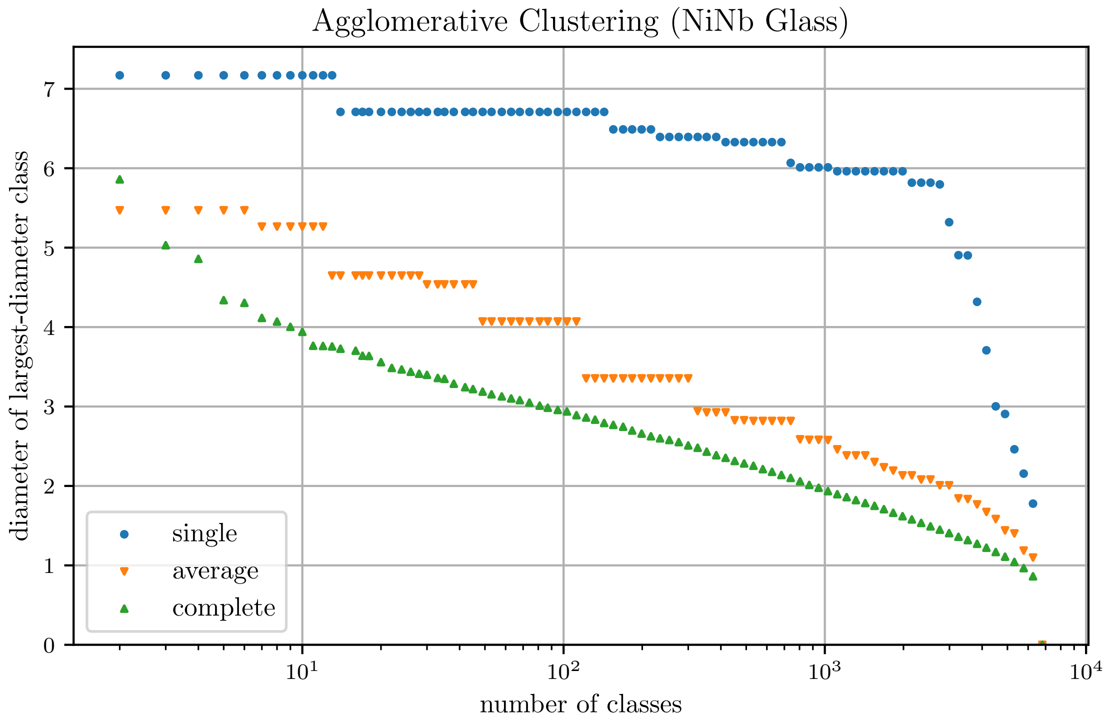

Supplementary Figure 4: Diameter of largest-diameter class as a function of number of classes for the 3d NiNb glass sample. Diameter of a class is the largest GIIP distance between members of the class. Partitioning this dataset into as many as 1000 classes still results in nearly two atoms (in GIIP distance units) of within-class variation. Source data are provided as a Source Data file.

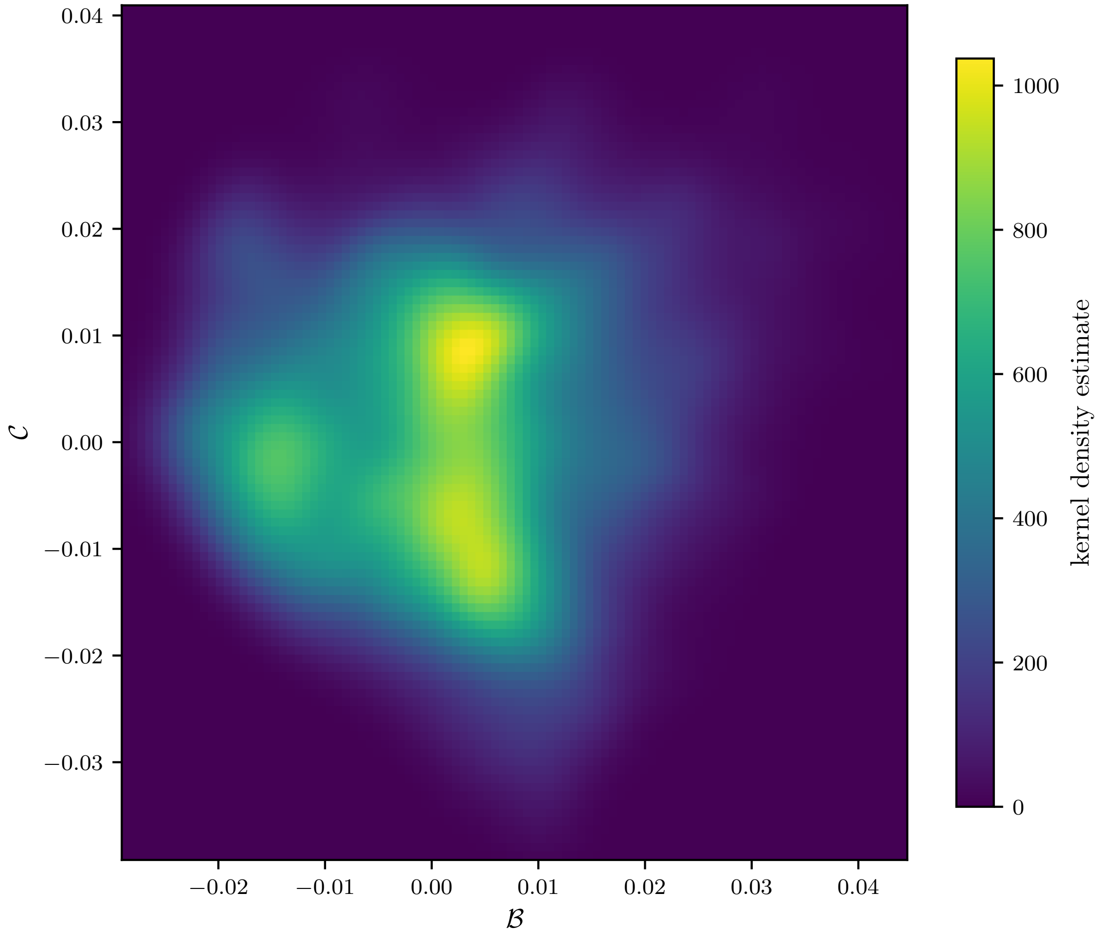

Supplementary Figure 5: Kernel density estimate of 3d NiNb glass LAEs in the ($\mathcal{B} \times \mathcal{C}$)-plane of the diffusion map. The multimodal distribution reflects energetically preferred regions of local structural space.

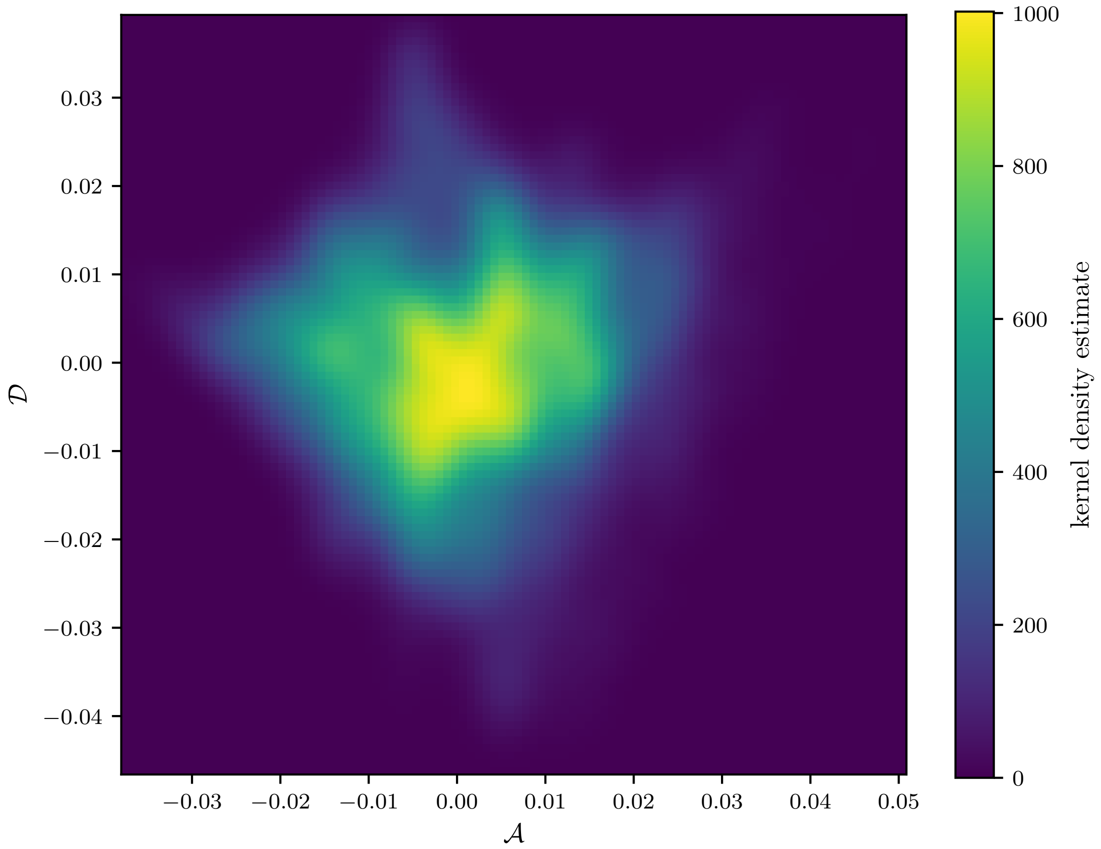

Supplementary Figure 6: Kernel density estimate of 3d NiNb glass LAEs in the $(\mathcal{A} \times \mathcal{D})$-plane of the diffusion map. This distribution is less obviously multimodal than the $\mathcal{B} \times \mathcal{C}$-plane.

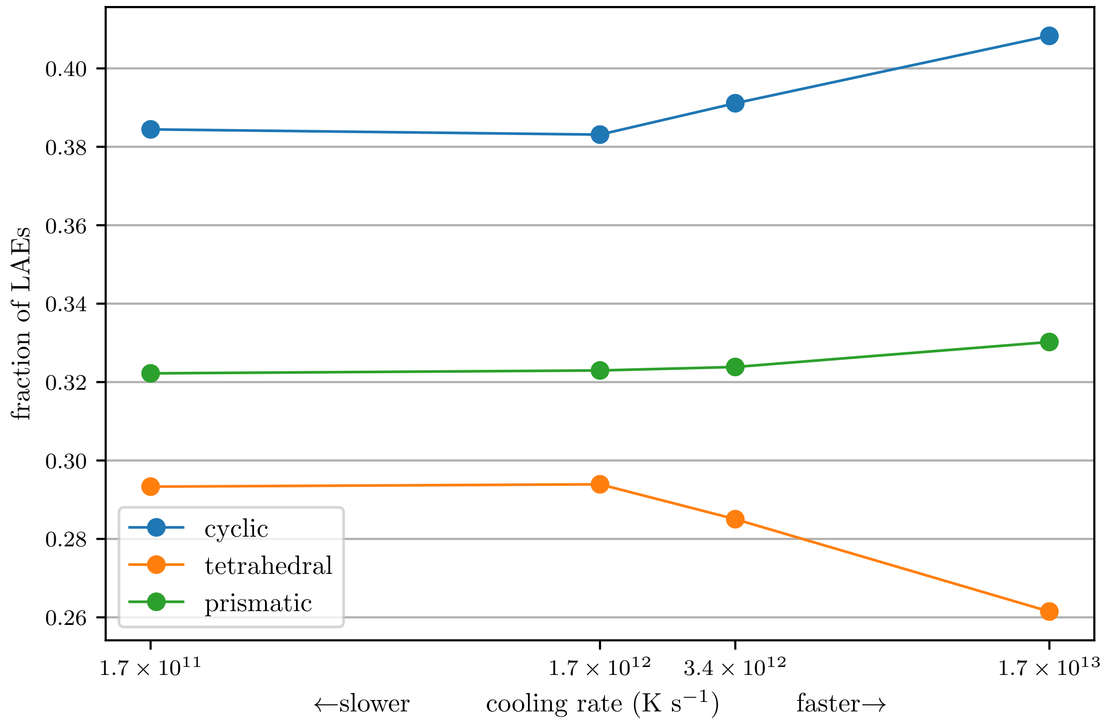

Supplementary Figure 7: Populations of cyclic, tetrahedral, and prismatic sectors of the $(\mathcal{B} \times \mathcal{C})$ plane as a function of cooling rate. These sectors are defined in the next figure. Source data are provided as a Source Data file.

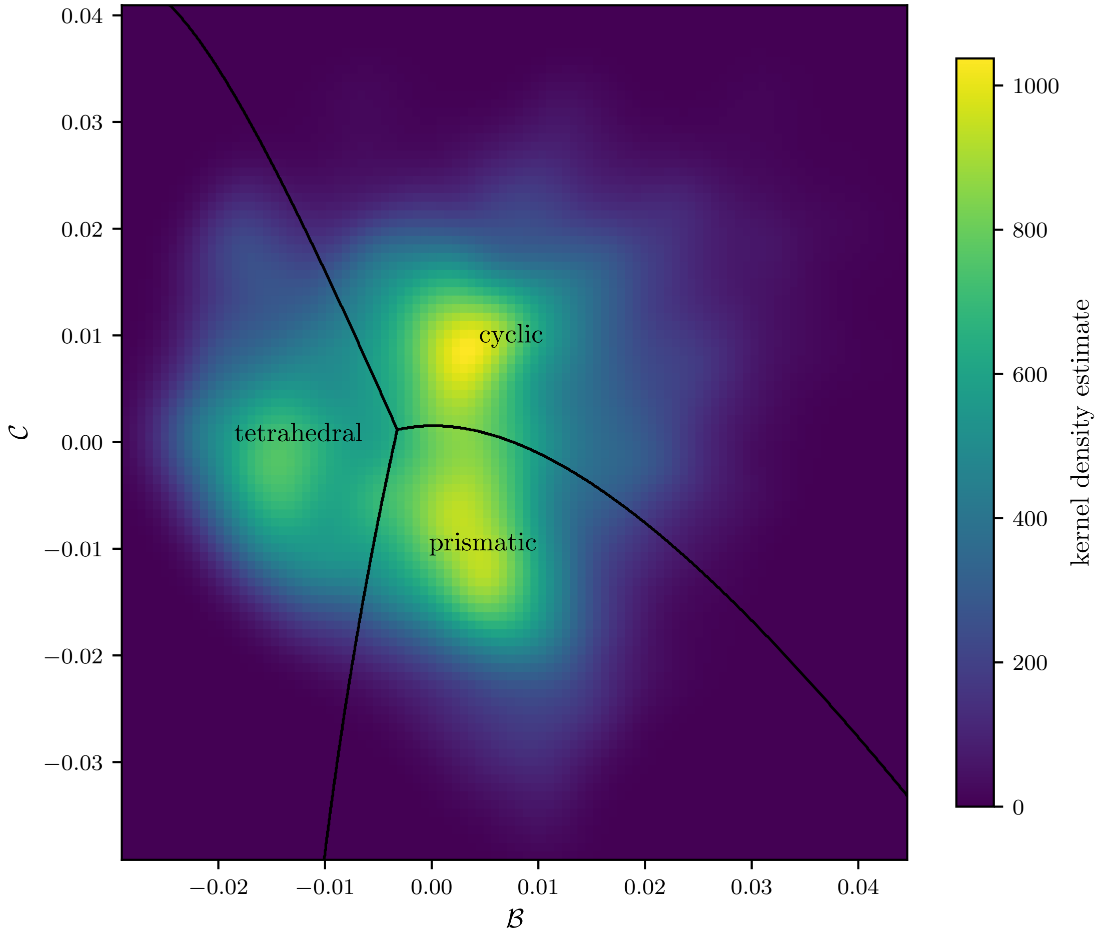

Supplementary Figure 8: Division of the ($\mathcal{B} \times \mathcal{C}$) plane into tetrahedral, cyclic, and prismatic sectors. The aggregated populations of these regions are shown in the figure above for the four different quench rates; regions identified using a 3-cluster Gaussian mixture model.
